# Successful Conservative Management of Ruptured Ovarian Cysts with Hemoperitoneum in Healthy Women

**DOI:** 10.1371/journal.pone.0091171

**Published:** 2014-03-07

**Authors:** Jee Hyun Kim, Seung Mi Lee, Ji-Hyun Lee, Yu Ri Jo, Min Hoan Moon, Jonghwan Shin, Byoung Jae Kim, Kyu Ri Hwang, Taek Sang Lee, Kwang Bum Bai, Hye Won Jeon

**Affiliations:** 1 Department of Obstetrics and Gynecology, Seoul National University Bundang Hospital, Seongnam, Korea; 2 Department of Obstetrics and Gynecology, Seoul Metropolitan Government Seoul National University Boramae Medical Center, Seoul, Korea; 3 Department of Obstetrics and Gynecology, Seoul National University Hospital, Seoul, Korea; 4 Department of Diagnostic Radiology, Seoul Metropolitan Government Seoul National University Boramae Medical Center, Seoul, Korea; 5 Department of Emergency Medicine, Seoul Metropolitan Government Seoul National University Boramae Medical Center, Seoul, Korea; Baylor College of Medicine, United States of America

## Abstract

**Study Objective:**

To determine the success rate of the “intended conservative management strategy” of ruptured ovarian cysts with hemoperitoneum and the risk factors for surgical interventions in healthy women of reproductive age.

**Methods:**

Patients who visited the emergency department with abdominal pain and were diagnosed with a ruptured ovarian cyst with hemoperitoneum between August 2008 and June 2013 were included in this retrospective study. The diagnosis of the ruptured ovarian cysts and hemoperitoneum was based on the clinical symptoms, physical examination and ultrasound and CT imaging. The rate of surgical interventions and the risk factors for surgical intervention were determined.

**Results:**

A total of 78 women were diagnosed with a ruptured ovarian cyst with hemoperitoneum. Most patients (80.8%, 63/78) were managed conservatively, and 19.2% of the patients (15/78) required a surgical intervention. In the multiple logistic regression analysis, the diastolic blood pressure (dBP) (odds ratio [OR] of 0.921 with 95% confidence interval [CI] of 0.855–0.993) and the depth of the total pelvic fluid collection in CT (DTFC_CT) (OR 1.599 with 95% CI 1.092–2.343) were the significant determining factors of surgical intervention after adjustment. The rate of surgical intervention was 6.5% vs. 15.8% vs. 77.8% in the patients with neither dBP≤70 mmHg nor DTFC_CT≥5.6 cm, those with only one of those features, and those with both, respectively.

**Conclusion:**

Most cases of ruptured ovarian cysts with hemoperitoneum can be managed conservatively. A low diastolic blood pressure and a large amount of hemoperitoneum suggest the need for surgical intervention.

## Introduction

Functional ovarian cysts are relatively common in women of reproductive age. Most functional ovarian cysts do not cause symptoms and resolve in 1 to 2 months with expectant management [1]. Although rupture and hemorrhage of an ovarian cyst may be physiologic events and a self-limited process, they may cause significant hemoperitoneum and require surgical intervention [2]. Because of these diverse clinical situations, a standard management protocol for ruptured ovarian cysts with hemoperitoneum is not well established.

Historically, ruptured ovarian cysts with hemoperitoneum have been a surgical emergency. Previous studies reported the rate of surgical intervention in this situation as high as 80% [3], [4]. Recent advances in imaging studies have enabled clinicians to make accurate early diagnoses and use careful expectant management with proper patient safety, and conservative management strategies have been suggested as alternatives to conventional surgical intervention [5].

There is a lack of information regarding the success rate of conservative management of ruptured ovarian cysts with hemoperitoneum, which can be performed especially in healthy women without coagulopathies. To address this issue, we evaluated the success rate of the “intended conservative management strategy” of ruptured ovarian cysts with hemoperitoneum and the risk factors for surgical intervention in healthy women of reproductive age.

## Materials and Methods

### Study Design

This retrospective study included patients who visited the emergency department of Seoul Metropolitan Government Seoul National University Boramae Medical Center with abdominal pain and were diagnosed with a ruptured ovarian cyst with hemoperitoneum between August 2008 and June 2013. The diagnosis of ruptured ovarian cyst and hemoperitoneum was made by clinical symptoms, physical examination and imaging findings on ultrasound and CT.

Cases with a positive urine hCG pregnancy test and those with suspected ovarian cyst torsion, endometriosis or ovarian bleeding after ovum pick-up were excluded. The clinical parameters and image findings were compared between patients who were managed conservatively and those who underwent surgery. This study was approved by the Institutional Review Board of Seoul Metropolitan Government Seoul National University Boramae Medical Center. Written consent was not obtained. Because it was a retrospective chart review, we had a difficulty to contact the subjects and we determined the risk to the study subjects is minimal. Patient records/information was anonymized and de-identified prior to analysis. All clinical investigation must have been conducted according to the principles expressed in the Declaration of Helsinki.

### Clinical Parameters and CT Image Analysis

Information of clinical parameters was retrieved by review of medical records. At the time of initial presentation at emergency department, patients were evaluated with vital signs, hemoglobin level, and ultrasound and CT images by attending gynecologist.

**Figure 1:**
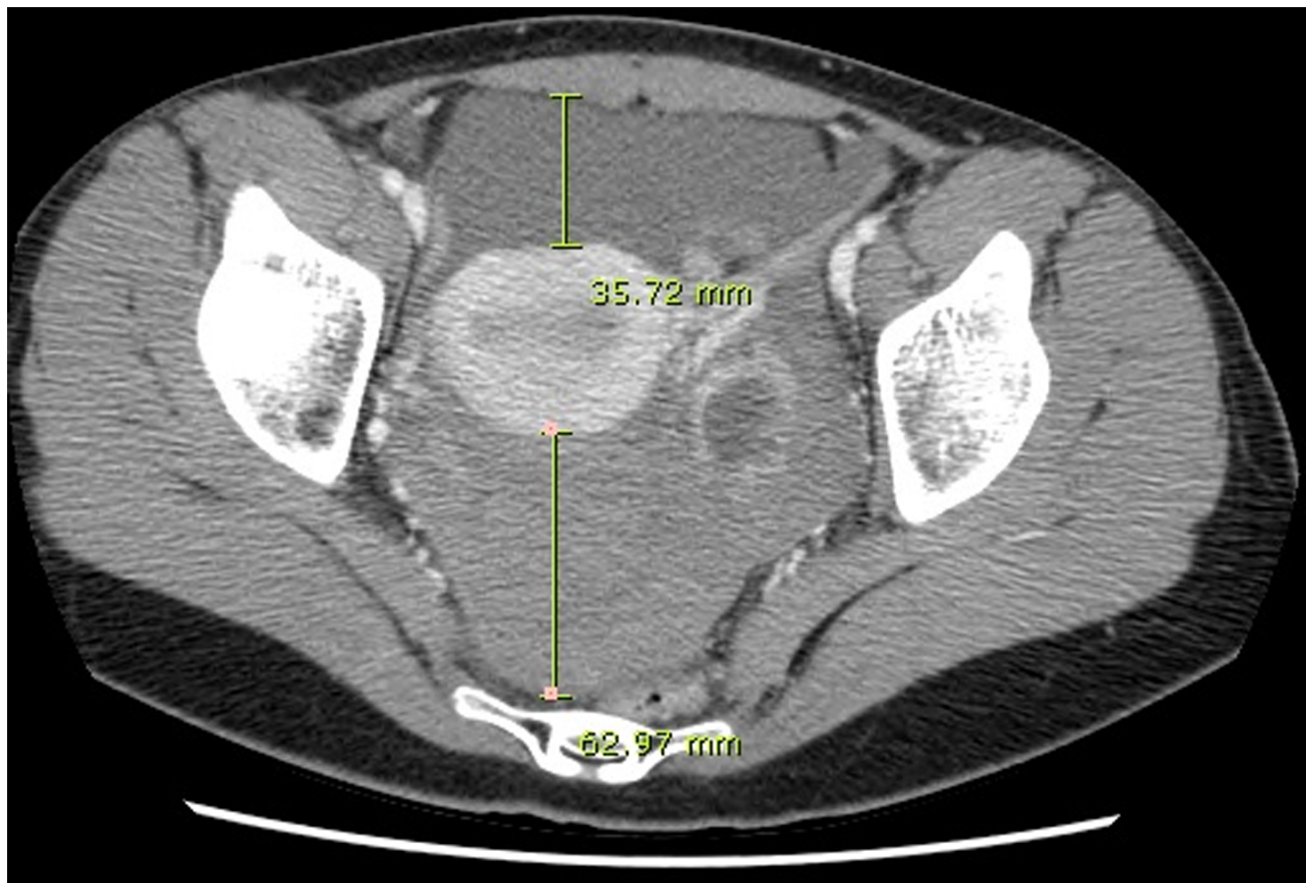
CT quantification of hemoperitoneum. The depth in the anterior cul-de-sac and the posterior cul-de-sac are defined the distance from the anterior peritoneum to the anterior uterine wall and the distance from the posterior uterine wall to the rectum, respectively.

One physician (KJH) retrospectively reviewed the size of the ovarian cysts and the amount of hemoperitoneum on the CT images. The size of the ovarian cyst was measured along its major axis. To differentiate hemoperitoneum from physiologic pelvic fluid, the mean CT attenuation values were measured by placing a round region of interest (ROI) in the pelvic fluid on the precontrast images. Pelvic fluid with CT attenuation >30 Hounsfield units (HU) was designated as hemoperitoneum [6]. The amount of hemoperitoneum was quantified by measuring the depth of the pelvic fluid at the level of the tubal isthmus connecting to the uterus in the axial plane of the CT images. The distance from the anterior peritoneum to the anterior uterine wall and the distance from the posterior uterine wall to the rectum were designated as the depth in the anterior cul-de-sac (ACDS) and the depth in the posterior cul-de-sac (PCDS), respectively (Figure 1). The total depth of hemoperitoneum was defined as the sum of the depth in the ACDS and the depth in the PCDS.

### Clinical Management of Hemoperitoneum

Conservative management strategy is the preferred management protocol in our institution for patients with a ruptured ovarian cyst and hemoperitoneum, especially in patients without coagulopathies. We usually recommend hospitalization for the close monitoring of vital signs, hematocrit levels, and repeat imaging to assess the possibility of active bleeding. The usual indications for surgical interventions are unstable vital signs, a significant hemoglobin decrease or increasing hemoperitoneum on a follow up imaging study, and severe or persistent abdominal pain despite use of the analgesics. The decision to perform a surgical intervention is at the discretion of the attending physician.

To evaluate the hospital course, the need for transfusion, the duration of hospitalization, and hemoglobin levels at discharge were also compared between the two groups.

### Statistical Analysis

The data were analyzed with SPSS software (version 20.0, Chicago, IL, USA). The data were compared by Fisher’s exact test or the Mann-Whitney U-test as indicated. A logistic regression analysis was performed. The result was considered significant when the P value was less than 0.05.

## Results

During the study period, 78 women were diagnosed with a ruptured ovarian cyst with hemoperitoneum on CT imaging. Most of the patients (80.8%, 63/78) were managed conservatively, and 19.2% of patients (15/78) required surgical intervention. All the patients who required surgical intervention were treated laparoscopically, and hemorrhagic corpus luteum was confirmed in all of the cases for which the pathologic results were available (n = 13).

Table 1 compares the clinical characteristics and CT image findings of the study population at initial presentation. There were no significant differences in the age, body mass index, marital status, parity, menstrual phase at the time of rupture, presence of antecedent to pain, initial pulse rate and hemoglobin level between the patients who required surgical intervention and those who were managed conservatively. The patients who required surgery had lower systolic and diastolic blood pressures at the initial presentation (110.7±10.7 vs. 118.9±15.3 mmHg, p = 0.051; 66.9±7.9 vs. 74.4±11.0 mmHg, p = 0.007).

**Table 1:** Clinical parameters and CT image findings of the patients at initial presentation.

	Surgery	Conservative management	P value
	(n = 15)	(n = 63)	
**Clinical parameters**			
Age (year)	25.7±3.9	26.2±6.4	0.760
Body mass index (kg/m^2^)	19.5±1.6	20.0±1.9	0.359
Married, n (%)	2 (13.3%)	14 (22.2%)	0.723
Nulliparity, n (%)	15 (100%)	53 (84.1%)	0.195
Menstrual cycle, n (%)			0.855
Follicular phase	0 (0%)	1 (1.6%)	
Luteal phase	11 (73.3%)	42 (68.9%)	
Undetermined due to irregular cycle	4 (26.7%)	18 (29.5%)	
Antecedent to pain, n (%)			1.000
Intercourse	7 (46.7%)	29 (46.0%)	
Others or unknown	8 (53.3%)	34 (54.0%)	
Initial vital signs			
Systolic blood pressure (mmHg)	110.7±10.7	118.9±15.3	0.051
Diastolic blood pressure (mmHg)	66.9±7.9	74.4±11.0	0.007
Pulse rate	87.1±13.2	87.4±15.5	0.694
Initial hemoglobin level (g/dL)	12.3±1.4	12.7±1.0	0.272
**CT image findings**			
Site of ovarian cyst, n (%)			0.149
Right ovary	12 (80.0%)	37 (58.7%)	
Left ovary or both ovaries	3 (20.0%)	26 (41.3%)	
Maximum diameter of ovarian cyst (cm)	4.1±1.2	3.9±1.2	0.368
PCDS fluid collection, depth (cm)	3.3±1.6	3.0±1.5	0.652
ACDS fluid collection, depth (cm)	2.9±2.2	1.5±1.4	0.011
Total pelvic fluid collection, depth (cm)	6.2±2.0	4.4±2.0	0.005

PCDS, posterior cul-de-sac; ACDS, anterior cul-de-sac.

Imaging findings on CT, there was no difference in the site (right ovary vs. left ovary or both ovaries) or maximum diameter of the ovarian cysts. The patients who required surgery had a significantly large amount of hemoperitoneum (the depth of total pelvic fluid collection in CT [DTFC_CT], 6.2±2.0 vs. 4.4±2.0 cm, p = 0.005).

A multiple logistic regression analysis demonstrated that the diastolic blood pressure (dBP) (odds ratio [OR] of 0.921 with 95% confidence interval [CI] of 0.855–0.993) and the amount of hemoperitoneum (OR 1.599 with 95% CI 1.092–2.343) were the significant determining factors for surgical intervention after adjustment (Table 2).

**Table 2:** Logistic regression results for predicting surgical intervention in patients managed for ruptured ovarian cysts with hemoperitoneum.

Parameters	Odds ratio	95% confidence interval	Significance
Nulliparity	119070908.740	0.0–inf	0.999
Diastolic blood pressure	0.921	0.855–0.993	0.033
Initial hemoglobin	0.821	0.428–1.576	0.553
Site of ovarian cyst (Right)	3.495	0.701–17.414	0.127
Total pelvic fluid collection, depth (cm) on CT	1.599	1.092–2.343	0.016

**Figure 2:**
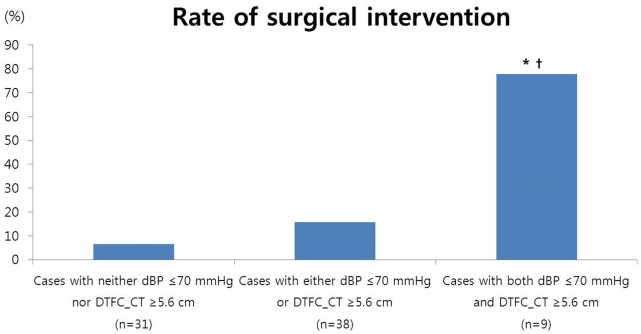
The rate of surgical intervention according to the absence or presence of risk factors. *, p<0.05 compared to the cases with neither a dBP≤70 mmHg nor a DTFC_CT≥5.6 cm; †, p<0.05 compared to cases with either a dBP≤70 mmHg or a DTFC_CT≥5.6 cm. The proportions were compared with Fisher’s exact test.

Receiver-operating characteristic (ROC) curves were obtained for the value of dBP and DTFC_CT using surgical intervention as the end point, a dBP≤70 mmHg and DTFC_CT≥5.6 cm were selected, and Figure 2 shows the rate of surgical intervention according to the absence or presence of these risk factors. The rate of surgical intervention in patients with neither dBP≤70 mmHg nor DTFC_CT≥5.6 cm was as low as 6.5%, while this risk increased in patients who had either of these risk factors and in those who had both of these risk factors (6.5% vs. 15.8% vs. 77.8%, p<0.001, chi-square for trend). Patients who had both of these risk factors had a significantly increased rate of surgical intervention compared to the patients who had none or one of these factors.

In the hospital course, patients who required surgery experienced a more rapid hemoglobin decrease over 4 hours (1.6±0.6 vs. 1.2±0.8 g/dL, p = 0.026), and needed transfusions more frequently than those who were managed conservatively (53.3% vs. 11.1%, p = 0.001). The duration of hospitalization was longer in patients with surgical interventions than those who were managed conservatively (4.1±0.8 vs. 3.1±1.4, p = 0.002) (Table 3).

**Table 3:** Hospital course of the patients.

Parameters	Surgery	Conservative management	P-value
	(n = 15)	(n = 63)	
Hemoglobin decrease over 4 hours* (g/dL)	1.6±0.8	1.2±0.8	0.026
	(n = 15)	(n = 54)	
Transfusion	8 (53.3%)	7 (11.1%)	0.001
Hospital stay (days)	4.1±0.8	3.1±1.4	0.002
Hemoglobin level at discharge (g/dL)	10.6±1.3	11.1±1.2	0.168

* Included hemoglobin follow up duration ≤10 hours only.

## Discussion

The principal findings of this study are as follows: 1) most of the patients who presented with acute abdominal pain caused by a ruptured ovarian cyst with hemoperitoneum could be managed conservatively; 2) the risk factors for surgical intervention were a low diastolic blood pressure at initial presentation and a large amount of hemoperitoneum; 3) the rate of surgical intervention in patients who had both of these risk factors was as high as approximately 80% in contrast to less than 10% in patients without these risk factors.

The rate of surgical intervention was relatively low in the present study; this is different from previous studies that reported a rate of surgical intervention as high as 80% [3], [4]. Ho et al. [3] reported that there were no patients treated medically in the 1980s, and 18.7% of patients treated medically in the 2000s of the women diagnosed with a ruptured corpus luteum and hemoperitoneum. There was an emerging trend towards conservative treatment from the 1980s to the 2000s, but surgical intervention remained the mainstay of treatment in the 2000s. A study by Raziel et al. [4] published in 1993 reported the rate of surgical intervention to be 83% in patients with a ruptured corpus luteum. They emphasized the value of ultrasound and diagnostic laparoscopy in deciding on the management, and eighteen patients (25.7%) underwent diagnostic laparoscopy with no further surgical procedures. Eighteen patients with concurrent ectopic pregnancy were included in Raziel’s study and contributed to the high rate of surgical intervention in that study.

The relatively low rate of surgical intervention in the current study is explained by two factors. First, recent advances and the frequent use of imaging modalities including CT could allow for the diagnosis of a different cause of a surgical abdomen and the avoidance of surgical intervention. A ruptured corpus luteum with hemoperitoneum was frequently misdiagnosed preoperatively as appendicitis, ectopic pregnancy, endometriosis, and neoplasm when CT was not commonly used [7]. The value of diagnostic laparoscopy was considered to be of great value in making the diagnosis when faced with equivocal or uncertain conditions. Second, our “intended conservative management strategy” of hemoperitoneum resulting from a ruptured ovarian cyst might contribute to the low rate of surgical intervention. We considered surgical intervention only when patients had unstable vital signs, a significant hemoglobin decrease or increasing hemoperitoneum on a follow up imaging study, or severe or persistent abdominal pain. Our indications are similar to those suggested by Bottomley: “1. Hemodynamic compromise, 2. Diagnostic uncertainty or likelihood of torsion, 3. No relief of symptoms within 48 hours of presentation, 4. Increasing hemoperitoneum on ultrasound or a falling hemoglobin concentration” [5].

In the current study, the significant risk factors for surgical intervention were a low blood pressure and the amount of hemoperitoneum, both of which can reflect the amount of current bleeding. There was no difference in the pulse rate between the women who required surgery and those treated with conservative management, and this finding is consistent with previous reports in which some women with acute hemoperitoneum and hypotension did not show tachycardia [8]–[10]. The initial hemoglobin level was not helpful in predicting surgical interventions, and this may be explained by the fact that the initial hematocrit may not reflect the acute blood loss. A hematocrit change was previously reported to be a reliable indicator of continuing blood loss [11], and in this study, the women who required surgery showed a more rapid hemoglobin decrease than those who were managed conservatively.

In this study, we included only patients who had CT findings at the initial diagnosis to eliminate the possibility of simple or physiologic peritoneal fluid and to quantify the hemoperitoneum objectively. Normal ovulation can also result in fluid collection in the pelvic cavity, and this may be confused with hemoperitoneum on ultrasound [12]. A CT scan may have further or extra diagnostic values over ultrasound [2], [13], [14] in terms of the differential diagnosis that includes other causes of a surgical abdomen, including appendicitis, and this allowed for the conservative management of ruptured ovarian cysts with hemoperitoneum with proper patient safety. In addition, CT has the advantage to evaluate the site and amount of bleeding. On CT imaging, we quantified the amount of hemoperitoneum as the depth of pelvic fluid. Another possible method to quantify the amount of hemoperitoneum may be the categorization of the amount of pelvic fluid as “none or trace, small (limited to the cul-de-sac), moderate (fluid around the liver, paracolic gutter, and pelvis), or large (additional fluid between the small bowel loops and colon)” [15], or the measurement of the volume using three dimensional CT, or the single deepest depth measurement. We adopted the total depth of the hemoperitoneum, which was defined as the sum of the depth in ACDS and the depth in PCDS at the level of the tubal isthmus. This simple and objective measurement can be easily used in clinical practice.

In the previous two studies reporting the management of ruptured corpus luteum, diagnoses were made without CT imaging, depending on the ultrasonography, laparoscopy and culdocentesis [4] or serial ultrasound examinations and positive response to empirical tranexamic acid therapy [3]. The diagnoses including CT imaging in this study might be one of the factors contributing the lower rate of surgical intervention, as discussed above. This is the first study to evaluate the management strategies of the ruptured ovarian cysts with hemoperitoneum diagnosed with CT imaging as far as we know.

A limitation of this study is its retrospective nature and that it was performed at one institution. A prospective, multicenter study should be followed to validate our findings. If a risk evaluation system is well validated, the outpatient management can be considered.

In conclusion, conservative management was successful in the majority of patients with ruptured ovarian cysts with hemoperitoneum. A low diastolic blood pressure and a large amount of hemoperitoneum suggest the need for surgical intervention. These findings could be helpful in decision making in clinical practice.
